# Current Concerns about Microplastics and Nanoplastics: A Brief Overview

**DOI:** 10.3390/polym16111525

**Published:** 2024-05-29

**Authors:** Marco Morreale, Francesco Paolo La Mantia

**Affiliations:** 1 Department of Engineering and Architecture, Kore University of Enna, Cittadella Universitaria, 94100 Enna, Italy; marco.morreale@unikore.it; 2 National Interuniversity Consortium of Materials Science and Technology (INSTM), Via Giusti 9, 50121 Firenze, Italy; 3 Department of Engineering, University of Palermo, Viale delle Scienze, 90128 Palermo, Italy

**Keywords:** microplastics, nanoplastics, recovery and recycling, environmental impacts

## Abstract

The widespread and increasing use of plastic-based goods in the present-day world has been raising many concerns about the formation of microplastics, their release, their impacts on the environment and, ultimately, on living organisms. These concerns are even greater regarding nanoplastics, i.e., nanosized microplastics, which may have even greater impacts. In this brief review, although without any claim or intention to exhaustively cover all the aspects of such a complex and many-sided issue, the very topical problem of the formation of microplastics, and the even more worrisome nanoplastics, from polymer-based products was considered. The approach is focused on a terse, straightforward, and easily accessible analysis oriented to the main technological engineering aspects regarding the sources of microplastics and nanoplastics released into the environment, their nature, some of the consequences arising from the release, the different polymers involved, their technological form (i.e., products or processes, with particular attention towards unintentional release), the formation mechanisms, and some possible mitigation pathways.

## 1. Introduction

The use and presence of polymers (more commonly referred to as “plastics”) is well known to be, nowadays, a necessary part of ordinary daily life. However, the exponentially increasing utilization of plastic items has been posing downsides and threats in terms of environmental impacts [1,2,3,4]. Due to unsatisfactory (and, in some cases, insufficient) recycling procedures and rates, and the well-known lack of biodegradability of traditional plastics [1,2,5,6], the persistence of plastic-based items leads to the formation of macroplastics, mesoplastics and microplastics, depending on the size and the source [7,8,9,10,11,12,13,14,15,16,17,18,19], with multiple and concerning environmental impacts, affecting both fresh and marine waters [20,21,22], soils [23,24], and air [20,21,24].

In more detail, macroplastics (>2.5 cm) and smaller mesoplastics (5 mm–2.5 cm) can come from different sources, such as direct and unintended release into the environment, while microplastics (and, in turn, nanoplastics) typically come from a combination of two primary pathways, i.e., direct and/or unintended release into the environment, and the multiple degradation phenomena that can affect not only the above mentioned microplastic and mesoplastics, but also from products such as paints and tires during their normal life cycle. It is estimated that, by 2060, global plastic leakage into the environment should double to approx. 45 million tonnes/year with subsequent accumulation of plastics into water bodies estimated to triple [25]. In general, it can be stated that microplastics have been found in terrestrial, marine and freshwater ecosystems as well as in food and drinking water, and their continuous release leads to several concerns related to the ecosystem, since exposure to microplastics has been linked to a series of negative ecotoxic and physical effects on living organisms [7,8,9,10]. 

As mentioned, microplastics released into the environment can come from multiple sources, but it is evident that one of the most obvious, and technically reducible, comes from intentional addition to several products such as cosmetics, detergents, paints, fertilizers, etc. 

There are, in summary, several mechanisms and sources involved, which in turn require accurate classifications and complicate the full understanding of the problems linked to the environmental release of these levels of pollutants, and the development of adequate strategies for containment. 

In this paper, therefore, we aim to provide a synthetic overview of the main aspects, from the currently available technical-scientific literature regarding the sources of microplastics and nanoplastics released into the environment, their nature, some of the consequences related to such release, with an approach specifically oriented towards the different polymers involved, their technological form (i.e., products or processes), the formation mechanisms and some possible mitigation pathways. This brief review is therefore oriented to the main technological engineering aspects and in no way aims at discussing and analyzing the issues related to human health, or in general the biological (e.g., effects on flora and fauna) and environmental (e.g., air, soil, water pollution) aspects, for which it is recommended to rely on specifically dedicated papers available in the literature (some examples of which are cited throughout the present manuscript).

## 2. Classification

In order to deepen the discussion about sources and countermeasures to microplastic-related pollution, it is first necessary to have a clear idea about nomenclature and classifications. 

The main classifications are typically based on the particle/fiber size (i.e., the larger between length and diameter) and the sources/mechanisms originating those particles. 

In more detail, the several mechanisms (direct and unintended release into the environment, united release due to degradation phenomena, etc.) and sources, as well as a classification based on the particle/fiber size (i.e. length or diameter, depending on which is the larger), may be summarized as shown in Table 1. 

With particular concern to microplastics, it can be observed that they can be directly released into the environment in their primary form (for instance scrubbers in cosmetics, liquid soaps, paints, abrasive products; pharmaceuticals; pellets; textile fibers; release of particles and fibers from production and maintenance operations of objects made of polymeric material) and in this case, they are usually referred to as “primary microplastics”; or they can come from macroplastics and mesoplastics (but also from primary microplastics) through degradation processes (thermal, thermo-oxidative, photo-oxidative, biological, etc.), mainly due to the action of the environment, but also from washing and drying of fabrics, or tire abrasion; in this case, they are usually referred to as “secondary microplastics”. There is also a difference in terms of intentionality of the release since some are intentionally added to a variety of products (e.g., fertilizers, plant protection products, cosmetics, industrial and household cleaners, cleaning products, paints, products used in the oil and gas industry, soft infill material on synthetic turf sports fields, etc.) while others are not intentionally generated, and therefore are not intentionally added to manufactured goods (e.g., those released by paints, tires, textiles and geotextiles, pellets and capsules for detergents, but also pellets dispersed during handling and transformation operations).

Furthermore, a significant threat comes from nanoplastics (i.e., characterized by nanosized length and/or diameter), which can come from different pathways, some similar to those described from primary microplastics and others similar to those regarding secondary microplastics, especially when fabrics washing and drying, or tire abrasion are concerned (see Table 1).

## 3. Sources and Concerns

### 3.1. Overview

Significant concerns have been raised about the various effects of micro and nanoplastics on human health [12,26,27,28], although there is no universal agreement on them, also because the reliability of in vitro models is somewhat questioned [29]. Nanoplastics, in particular, have been shown to have the ability to bioaccumulate and invade the food chain [12,30,31]. While there are very limited human data, there is evidence from animal studies, but most of them focus only on specific types of micro- and nanoplastics, while some common types remain substantially uninvestigated; furthermore, there is still a lack of standardized methods for correct definition and detection of microplastics (MP) and nanoplastics (NP) [27,32]. In brief, it can be stated that methods and techniques to detect, identify, and monitor microplastics and nanoplastics include gas chromatography-mass spectrometry (GC-MS), Fourier-Transform Infrared (FTIR) and Raman spectroscopy [12]. Several methods of detection, identification and quantification of MP and NP are available and are accurately reported, along with their limitations, in a review by Cai et al. [33], to which it should be referred for further details. Furthermore, the literature reports some new proposals for detection and identification such as, for instance, hyperspectral stimulated Raman scattering (SRS) imaging platform with automated plastic identification algorithms [34], a combination of micro-FTIR with Machine Learning [35] and even techniques for removal, such as multifunctional MXene-derived oxide microrobots [36]

According to a recent publication by the European Commission [33], the EU target is to reduce the release of microplastics into the environment at a “30-30” rate, i.e., 30% by 2030 (in comparison to the current situation). The target is ambitious since the world plastics leakage in 2060 is expected to double the one that occurred in 2019. The measures planned to obtain such goals are manifold, such as increasing the reuse and recyclability of packaging, the recycled plastic content and the use of biodegradable plastics, especially in specific applications (such as, for instance, mulch films). 

Accordingly, the very recently approved (October 2023) 2023/2055/UE Regulation has banned the sale of microplastics as such, of products where microplastics have been intentionally added and also those that can release microplastics during their use. The limitations concern widespread products such as cosmetics, detergents and filling materials for artificial sports surfaces. However, there are some significant products that are currently excluded from the sales ban, such as products that contain microplastics but do not release them, or their release can be reduced to a minimum (such as construction materials); products used in industrial sites; products already regulated by other EU dedicated regulations; products where microplastics have not been added on purpose but are present unintentionally (for instance, sludge and compost).

Furthermore, the previously mentioned EU publication [37] provides interesting figures about the release of microplastics (both primary and secondary) in the EU-27 countries. The main sources of unintentional release of microplastics are (in order) paints, tires, plastic pellets, textiles, geotextiles, and plastic detergent capsules, with paints, tires and pellets being the vast majority of the total. It may seem surprising that among the top six sources of microplastic pollution, those coming from packaging films or liquid containers are not directly present (see Table 2) the presumed quantity of which therefore seems at least less than 4000–6000 tons/year in 2019. 

Obviously, the possible generation of microplastics from these sources could only come from the highly degrading action suffered by these products when released into the environment. Actually, films and containers could undergo subsequent fragmentation processes due to mechanical and photooxidation actions and thus experience significant size reduction. At the same time, it should be noted that despite the often-widespread impression among public opinion that microplastic pollution is only associated with bags, envelopes, containers, etc. it should be also observed that, while these sources are certainly of concern with regard to seawater and shores [38] the above reported figures show that, as far as the whole environment (at least, in the EU) is concerned, there is a more important and threatening source to be taken into account. This, of course, does not mean that these sources are negligible, since microplastics and, likely, nanoplastics may come from them by fragmentation (although there is still a lack of a rigorous and widely accepted methodology to detect NPs in the environment) and, in any case, they can act as vectors for the transport of contaminants [39], but their role seems to be, in some cases (at least in the EU), mitigated.

Furthermore, it should be observed that a recent publication by the Italian agency of the World Wildlife Fund [40] states that 80% of the microplastics polluting the oceans come from rivers, and the majority does not come from large rivers (such as the Yang-Tze) but, instead, from a number of smaller rivers, such as the Pasig (Philippines) the Klang (Malaysia), the Ulhas (India) and the Sham Chun (China). Moreover, rivers from the tropical regions pour plastics into the oceans throughout the entire year, while those from temperate zones mainly during August. In both cases, the release of microplastics mainly depends on the proximity to large, densely populated areas/cities, especially when landfills and disposal sites are closer than 10 km to the river and increasingly on increasing the rainfall rate of the site. With specific concern to the Mediterranean Sea, according to the same source, more than 700 tons of plastics daily end up in the sea, and Italy (mainly through the Po river) is in third place after Turkey and Spain. However, in every case, the problem dramatically depends on the degree of implementation of suitable disposal and recycling practices of plastic items. Therefore, not just a simple reduction in the use of plastics, but especially suitable practices in the recovery, reuse and recycling of plastics, would help in mitigating the problem to a quite significant extent; another possible way (which can be seen as an alternative or, at least, as a complementary way) might be based on the use of biodegradable plastics (additional discussion will be given in a following section of this paper). 

Focusing on the main sources of unintentional release, the leakage from paints (which contain almost 40% of polymers, for durability and flexibility purposes) mainly occurs during removal or is due to tear and wear. The main sources are the marine, architectural and general industry sectors. 

Another important source comes from the friction of tires of passenger cars and trucks on road surfaces. Although the number of passenger cars is way higher than that of trucks, the proportional contribution of the latter is higher, due to weight. The problem may be partially addressed within the framework of future EURO-7 regulations, on the basis of actual affordability of some solutions beyond drastic measures such as limitation of individual transport: periodical compulsory tire alignment check, inflation pressure check, and decreased speed limits. 

As regards pellets, these are a typical source of primary microplastic leakage, and are highly contaminating since they can be easily transported by air and water, and also affect the soil, and can be ingested by a number of animal species (turtles, birds, fish, etc.), leading to harm and/or death. The EU is therefore working on a regulation setting mandatory requirements for the manufacturing and handling of plastic pellets. This is particularly important since it is reported that most of the typical pellets found in seawater (e.g., polypropylene, polyethylene, and polystyrene) can actually absorb harmful chemicals, such as polychlorinated biphenyl(PCBs), dichlorodiphenyltrichloroethane (DDT), and polycyclic aromatic hydrocarbons (PAHs) [41]. Furthermore, reducing plastic pellet leakage could be beneficial in terms of smarter use of energy resources. 

However, one of the most insidious and underestimated sources comes from synthetic textiles. They are capable of dispersing microfibers into water and air during their entire lifecycle, but especially during washing and drying. Solutions may be represented by compulsory measures regarding filters to be used for water and air outlets from washing machines and tumble dryers, and for the handling and disposal of their waste residues; development and/or increase in the use of biodegradable synthetic fibers; compulsory regulations imposing on producers to take responsibility about products before becoming waste, such as take-back plans. These improved recovery routines, in addition, may help in a smarter use of resources, in particular energy and raw materials. The same could occur if suitable policies were taken to promote (and make progressively mandatory) the implementation of particularly gentle washing programs (e.g., with the impossibility of exceeding certain temperature and speed thresholds) to be used specifically for synthetic fiber garments and educating users accordingly. Other solutions such as widespread limitation in the use of synthetic textiles seem to be rather utopistic, since the increasing world population is expected to require an increased amount of clothes and footwear of approx. 60% by 2030.

Overall, paints, textiles and tires seem to be the most threatening source, not only because of their overall emissions [37] but also because it seems reasonable [12,17,42,43,44] that the microplastics coming from their lifecycle may eventually lead to the formation of “secondary” nanoplastics. 

### 3.2. Paints

With regard to the leaking from paints and coatings, according to the CEPE (European Association of Producers of Paints, Printing Inks and Artists’ Colours, Brussels, Belgium), in order to minimize the release of microplastics, the main ways are basically limited to reducing the release of wet paint to the sewer, and to suitably collecting paint flakes during renovation operations [45]. In the same document, it is claimed that during the normal use of paints, release may occur only in two following scenarios: (I) cleaning of brushes or rollers under the tap; (II) landfill of left-over paint. It is added that the former is relevant only when a water-based dispersion paint is used and, at the end of a paint job, the brush or roller is washed through the drain; for this reason, CEPE suggests different methods for safely cleaning brushes after use. On the other hand, the latter occurs when painters do not dispose of the containers with left-over paints to suitable collection points, or when the local government does not offer such collection points. However, there could also be a third scenario: release of paint-film particles due to wear and tear (mainly related to environmental agents such as sunlight, rain, abrasion, etc.) of outdoor painted surfaces (which depends on the quality of the paint used), or to human-caused sanding of old paints before applying new layers of fresh paint; in this case, once more, it is a behavioral problem which may be significantly reduced if proper containing measures were taken into action. 

### 3.3. Tires

With particular concern to tire road wear particles (TRWP), there is a significant number of reports in the literature addressing this issue and to be cited [42,43,46,47,48,49,50,51,52,53,54,55,56,57,58,59,60,61,62,63,64,65,66,67,68,69,70,71,72,73,74]

**Figure 1:**
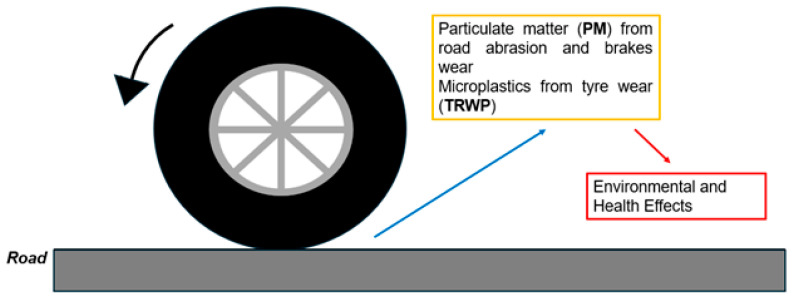
Schematic representation of TWRP generation and impacts. Adapted from [43] (under the terms and conditions of the Creative Commons Attribution CC BY license).

TRWPs are considered to be currently underrepresented in the assessment of actual pollution coming from microplastics. TRWP, as previously mentioned, come from the normal abrasion of both tires on road surfaces during various phases of driving (Figure 1), and they rise concern since they contain both natural and synthetic rubber, have been estimated to contribute up to 10% of the total plastic pollution of the oceans [43] and, considering the increasing rate of vehicle circulation worldwide, the concern should further increase since it is estimated that tires release about 6 million tonnes/year globally, and up to 12% of their mass to the environment over a typical lifespan [43,45], with an estimated average per-capita yearly release of TRWPs between 0.2 and 5.5 kg [45] and concerns rising from the possibility to be inhaled from humans due to the size of some of their fractions (up to 10% of the total), their mass distribution which is usually bimodal, with peaks at around 20–200 µm and at 2–10 µm [43], and the hazardous chemicals typically incorporated into tires including metals and organic additives [47,48], which may even include halogenated cyanoalkanes [49]. Further concerns come from the fact that techniques to correctly quantify their presence in the environment (especially in the air) are still under development and/or lacking standardization and/or being monitored to evaluate their actual accuracy [42,51].

Over recent years, some interesting papers have been published analyzing this microplastic pollution source [43,50,51,52]. The estimated per capita global average emission is about 0.81 kg/year, with emissions from car tires estimated to be significantly higher than those of other sources such as airplane tires (2% in comparison to total emissions from car tires), artificial turf (12–50%), brake wear (8%) and road markings (5%). It should be observed that the rough 5–10% contribution to ocean pollution makes TWRP a significant source such as fibers released from clothing during washing [75]. As for TWRP released into the air, not only it is difficult to separate them from other sources such as brake and road wear particles but is also hard to quantify human exposure in a realistic manner. It is estimated that TWRP contributes to about 3–7% of the environmental PM2.5 fraction; moreover, contribution to PM10 is estimated up to 11% [50]. From the above-cited studies [43,50,51,52], several considerations can be drawn. For instance, although there is a limited number of reliable data and different protocols and methodologies, a mean value of 110 mg/km or 68 mg/km/t for passenger cars could be derived, with the PM10 to total abrasion ratio around 2.5%, and the PM2.5 to PM10 ratio calculated around 40%. It was also found that the abundance of this pollutant is (as expected) closely related to road proximity; moreover, it is significantly influenced by both driving behavior and traffic density. While some more studies exist reporting data of accumulation alongside road environments, there is little information about the behavior of TRWP in aquatic environments, especially in conditions different from those of rivers, and therefore there is much uncertainty regarding TWRP paths into the marine environment and there are not many studies on the presence of TRWP in marine sediments. Furthermore, there is not much data on transport and degradation in soil. Health risks mainly concern inhalation, while there are practically no data about accumulation and exposure in the food chain. In summary, much work is still required to improve detection methods, assess actual environmental and health effects, and find practical measures in order to reduce TWRP emissions. The latter may include regular maintenance of vehicles (tire alignment, inflation pressure, etc.) and roads, encouraging the use of lighter vehicles (in this perspective, switching to heavier electric vehicles may constitute a problem), implementation of stricter speed limits, research on tire materials and design to increase wear resistance and durability (a very complex issue, which must take into account a number of factors; for instance, wear resistance may be increased by suitably using silica nanoparticles [76]), improving and encouraging use of public transportations, street cleaning, enhancement of roadside vegetation, etc. At the present time, as partially mentioned before, the only regulations regard polycyclic aromatic hydrocarbons (PAHs) in oils during tire production and the proposal for tire abrasion rates in the next Euro 7 regulations (which is still provisionary and some years to become operational). Tire abrasion rate limits should be beneficial in order to urge the research for more advanced tires, in which the lower per kilometer emission of particles would be matched by a reduction in fuel consumption, with very positive effects in terms of saving energy resources (both in terms of the lower fuel consumption but also an increased average tire life).

### 3.4. Textiles

As previously explained, an important and threatening source of microplastics (and nanoplastics) is represented by textiles [77]. Also, in this case, there are not yet fully standardized and accepted analytical methods for the detection, measurement and therefore classification of these microplastics [78]. However, it is believed [79] that synthetic textiles account for approx. 35% of total microplastics in the oceans; it was also found [80] that a single cloth can produce more than 1900 fibers per washing machine load. It is widely accepted that these microfibers can easily reach and even surpass wastewater treatment plants, also due to their lower biodegradability in comparison to natural fibers [81]. Furthermore, some estimates [82] state that up to 90% of microplastics in aquatic environments may be due to synthetic fibers; in the same source, it was also stated that among synthetic fibers, polyester fleece shed the highest amounts of microplastics (on average, almost 7500 fibers/m^2^/L per wash). For sure, all of the main synthetic fibers (polyesters, nylons, acrylics) shed microfibers on washing, but also the other processes such as production, packaging and transportation of synthetic fibers contribute to this shedding, although washing seems to be the main source, especially in the case of more processed textiles [83].

### 3.5. Degradation and Fragmentation

As briefly mentioned earlier, most secondary microplastics (and nanoplastics) come from fragmentation/degradation phenomena occurring on polymer-based items. In the following, therefore, a brief discussion is provided about the main degradation pathways of polymers. 

**Scheme 1:**
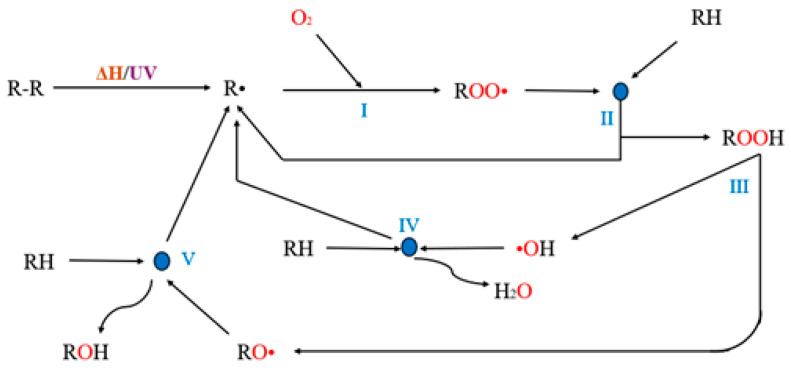
Photo-oxidation pathways.

First of all, it should be pointed out that several factors affect the degradation of plastics, such as intrinsic physico-chemical properties of the polymer involved (molecular weight, crystallinity, hydrophobicity, additives, structure) and environmental factors (temperature, oxygen and water presence, sunlight) [84]. In more detail, the energy required to revere the polymerization reactions and lead to polymer chain breaking can come in the form of heat, mechanical stress or solar radiation. Furthermore, biodegradation may also be taken into account, but it involves completely different driving forces (coming from microbial activity). A general scheme of polymer degradation cannot be proposed, since the degradation processes depend on the polymer and, in some cases, on the type of energy that causes the degradation, as well as the environment where the degradation occurs. For instance, in the case of polyolefins, the degradation process can certainly be considered to begin with the breakdown of the macromolecular chain via a radical mechanism. In the presence of oxygen, and therefore in the environment, the radicals react preferentially with it. Reactions with oxygen therefore cause the oxidation of macromolecular chains and this mechanism is particularly dangerous given that not only do macromolecules break and new chemical compounds are formed with oxygen, but also because during these reactions, new radicals are formed that re-enter the cycle, thus sustaining new oxidation reactions (see Scheme 1).

The R• radical reacts with oxygen, forming the oxygenated radical ROO• which, by extracting a hydrogen atom from another polymer chain, produces a hydroperoxide (ROOH), which, however, is highly unstable and thus decomposes into the two radicals RO• and OH•. The first, by extracting another hydrogen, closes the chain with the formation of an oxygenated macromolecule ROH and the OH• radical returns to the cycle creating a new macromolecular radical (see Scheme 1). Ultimately, while the polymer chains degrade, radicals are also formed regardless of the presence of solar radiation and therefore the degradation process continues. Therefore, molecular weight reduction is not the only effect of degradation; other phenomena can occur on the basis of the cause of degradation (heat, radiations, etc.), the presence of external agents (oxygen, chemicals, etc.) and the polymer structure. Depending on such external cause, four different types of degradation phenomena can be considered: (I) Thermal degradation and thermo-oxidation, due to heat and oxygen; (II) thermo-mechanical degradation, due to mechanical stress during processing; (III) photo-oxidation, caused by sunlight and oxygen; (IV) biodegradation, due to the metabolism of living organisms. Moreover, those that can actually lead to fragmentation are mainly photo-oxidation and biodegradation. Degradation due to solar radiation is possible because the energy of the ultraviolet (UV) component present in solar radiation between 290 and 400 nm is sufficient to cause the breaking of the macromolecule bonds. The energy radiated in this region of the spectrum, in fact, is approximately 60–100 Kcal/mol which is the typical bond energy of most polymers. Many polymers, including polyolefins, do not absorb UV radiation in the spectral range of this energy and therefore should not degrade; however, it is proven that even these polymers actually degrade under sunlight because of catalytic residues or functional groups formed during the processing and transformation stages. or even during storage. The most active groups for the initiation of photodegradation (chromophores) are the carbonyl C=O and peroxy -OOH groups, absorbing radiation between 290 and 400 nm and therefore being the starting points of degradation reactions. It is well known that, as the irradiation time increases, the value of the elongation at break decreases until the polymer becomes brittle. In these conditions the film breaks easily if subjected to even low stresses, generating small fragments and therefore microplastics. However, since UV rays do not have a strong penetrating power and typically exert their action only on a very thin layer (tenths of a millimeter) of the material, the effects of photo-oxidation are significantly dependent on thickness, affecting thinner films. 

In addition, a particular kind of degradation typically affects polycondensation polymers (such as polyesters, polyamides, polycarbonates), i.e., hydrolytic degradation. This is because, during polymerization, water molecules are expelled and must be removed so that these equilibrium reactions proceed in the direction of polymer formation; however, during high-temperature processing, the presence of humidity can shift the equilibrium towards depolymerization and therefore towards polymers with increasingly lower molecular weight and thus leading to embrittlement of the obtained materials. 

From this brief discussion about polymer degradation mechanisms, it is clear that those which can actually lead to the formation of microplastics in the environment are, mainly, related to photo-oxidation (which is related to fragmentation), although mechanical abrasion cannot be excluded. 

In summary, the role of photooxidation and fragmentation seems to be the most important. The two phenomena are, indeed, part of the same macro-phenomenon, since (as previously explained) photo-oxidation of plastic wastes leads to their embrittlement, which in turn leads to fragmentation, which in turn allows easier photooxidation of the inner macromolecules of each plastic fragment, and thus further fragmentation, and so on resulting in the formation of microplastics (and, eventually, nanoplastics). 

In a recent review study, Dimassi et al. [84] specifically focused on the degradation and fragmentation phenomena in seawater environment. The main aspects to be highlighted regarding the effects of degradation, resulting in a decrease in the molecular weight and modifications in the mechanical and other physico-chemical properties, and ultimately to the fragmentation phenomena, potentially even more dangerous since additives embedded in the polymer-based wastes could leach out, resulting in increased pollution threats. 

It was found from several literature sources that, in the marine environment, plastics can undergo degradation and fragmentation, and that this can occur to different extents and rates depending on the different plastic types and sizes of the items. However, it was also found that (as expected), UV-induced degradation occurs faster in on-shore conditions rather than in seawater. This makes it even more difficult to develop reliable models for plastic degradation in shore and sea environments, and further studies are needed.

In order to reduce the polymer pollution coming from degradation-fragmentation (especially in marine environments), the previously cited literature suggests, in summary, primarily relying on minimizing the production of terrestrial plastic waste by acting on public awareness and information regarding proper disposal; improving polymer waste collection and recycling systems; reducing/eliminating single-use plastics and, in general, primary microplastics (such as micro-scrubbers). 

### 3.6. What about Biodegradable Polymers?

Recently, some concerns have arisen also regarding the behavior of biodegradable plastics (BP) in comparison to conventional plastics (CP) in terms of micro(nano)plastics release. Most of the concerns arise from the consideration that BP should lead to microplastic formation more quickly than CP [85,86] and this should be especially troublesome in soil environments, but also in air [87]. Concerns regard, for instance, the potential impact of the release of various additives and/or fillers, the individual response of soil organisms can be largely diversified, and also the potential absorption (and following release) of pollutants from the soil is questioned such as, for instance, in the case of disposal of BP mulch films after use [88]. However, it is also explained that only a few data are available regarding the occurrence of soil microplastics from BP (basically, PLA and LDPE) and therefore further investigation is needed, focusing on more types, sizes, and concentrations of BP [85]. In more detail, a very recent review [86] states that microplastics from BP are more toxic than those from CP because they may release additives and absorb pollutants, but it also concludes that further studies are needed to fully characterize the formation and impacts of microplastics from BP, their actual behavior in comparison to CP with particular concern to soil organisms, the effects under actual field conditions rather than under laboratory conditions, the effects on plants and actual risk assessments on the food chain. While studies exist regarding several BPs other than PLA in actual environmental conditions, which provided confirmation of the potential, massive release of microplastics from some BPs and their interactions with living organisms, they also confirm that more investigation on actual ecotoxicity and environmental risk evaluation is needed [31,87,89], as well as more focused and “real world” investigations, without the typical (and sometimes rather rough) limitations and simplifications such as, for instance, the use of pristine plastics under highly controlled conditions, or the investigation on only one material at a time, or the use of pots and aquariums [31]. In addition, it should be observed that there is still only a limited number of papers investigating the interaction of BioMPs with heavy metals, an issue of relevant importance that should definitely be investigated in depth [31].

Furthermore, as already partially applies to microplastics from CP, in the case of BP microplastics there is an even greater lack of analytical methods for their measurement in soil, and therefore quick and suitable methods should be developed and validated (suggesting, in particular, thermoanalytical approaches as possible candidates) [90].

However, there is also literature data considering the above-summarized concerns as over-estimated. For instance, a model has been proposed that introduces the MPEP parameter, i.e., the potential of a polymer material to add persistent microplastics to the environment, which takes into account not only the production of microplastics via fragmentation, but also its disappearance (or, eventually, no disappearance, i.e., persistence) via biodegradation; in other words, it is proposed as the way to estimate microplastics build-up as the dynamic result of fragmentation reactions (which lead to the formation of microplastics) and biodegradation reactions (which remove microplastics) [91]. It was found that HDPE had an MPEP more than 1000 times higher than cellulose or a biodegradable commercial polymer. 

Furthermore, in a very recent publication, Degli Innocenti [92] openly criticized the “hype, hyperbole and publication bias” regarding the concerns over biodegradable mulch films, reporting studies performed at the mesocosm level and claiming that they were raising overestimated concerns about biodegradable mulches, since very high concentrations (up to 700 times the “normal” application doses) were used, quite unlikely to be experienced in usual agronomic practices, and thus urging to develop new and widely acceptable guidelines on the communication of the findings regarding biodegradable plastics, in order avoiding unjustified concerns in the public opinion.

### 3.7. Additional Concerns and Possible Workarounds

Based on the previously discussed issues, the most far-sighted solution to be taken quickly seems to be the improvement of collecting and recycling techniques for polymer-based items. 

Furthermore, it should be noted that the actual degradation environment can significantly affect the degradation behavior. For instance, in a recent study [93], photo oxidation of PP in three different environments (air, distilled water, sea water) and two different temperatures was investigated. It was found that the photo-oxidation kinetics were far slower in water (especially, in sea water) and significantly decreased on decreasing the temperature, due to the significant differences in oxygen availability (indeed, quite small in the two aqueous media, especially at higher temperatures because of the decrease in oxygen solubility, similar in both of them). 

An interesting result is related to the different biodegradability of bio-based polymers in different environments. In fact, as far as biodegradable polymers are concerned, and different from the fossil-derived polymers discussed so far, it would be expected for them to attain complete conversion to carbon dioxide and water in a reasonable time. However, it has been recently found [94] that at least some of them undergo low biodegradation rates in seawater due to limited oxygen availability; this should lead to slower photo-oxidation and therefore to longer persistence of plastic debris in water but, on the other hand, to slower fragmentation and thus microplastics generation. 

With specific regard to the even more threatening nanoplastics, in a recent review, Cai et al. [33] reported several studies regarding the state-of-the-art methodologies for pretreatment, separation, identification, and quantification. They found that, although most of the studies succeeded in detecting standard reference nanoplastics presence in environmental samples, they failed in separating and quantifying the nanoplastics from real field samples (i.e., from seawater, snow, air, sand, and agricultural soil) while only five analyzed real field samples. They confirmed (beyond the previously highlighted lack of widely standardized methods) the occurrence of limitations regarding the current spectroscopic techniques, since they are time-consuming, cannot provide a full covering of the entire nanosize range, are often destructive and still raise concerns about their actual reliability since there is often no confirmation of the chemical nature of detected polymers; therefore, it is concluded that more efforts should be carried out to improve the reliability and accuracy of nanoplastics detection and analysis from environmental samples.

## 4. Conclusions

In this brief review, although without any claim or intention to exhaustively cover all its aspects, the very topical problem of the formation of microplastics, and the even more dangerous nanoplastics, from items and products made of polymer-based materials was considered. It is clear and undeniable that the abuse of plastics, especially in mass, low-cost and short-life applications, is one of the main factors responsible for the environmental pollution from microplastics. Therefore, it would be essential to reduce or eliminate the use of plastic materials in applications such as detergents, cosmetics, pharmaceuticals, and, in general, to intervene as much as possible on the primary sources of microplastics (such as for example the production of synthetic fibers) and enact more stringent rules and procedures for the management of pellets during production, in order to avoid their release into the environment. On the other hand, it has been also found that important sources of microplastic pollution are secondary (and unintentional) ones, such as the washing of synthetic fiber garments, but even more the wear of tires and paints. In particular, it was also found from the literature that tires, textile fibers and paints seem to be among the main sources of nanoplastics, which are the ones that lead to the greatest concern in terms of possible effects on human health. These sources, as described above, seem to be much more significant than those that are more part of the collective imagination, such as packaging, films and their relative fragmentation/degradation; it is, however, true that some microplastics come from these products, and that nanoplastics through fragmentation of those microplastics can be formed. It is therefore necessary to intervene by improving the wear resistance of the polymeric materials used for these applications, and consequently, the quantity of micro/nanoplastics released for the same use; in the case of tires, for instance, this may also lead to significant advantages in terms of reducing energy and raw materials consumption. However, in all the cases, significant effort is also needed regarding the development and validation of reliable and widely accepted analytical techniques for the determination of actual microplastics and nanoplastics presence and amounts.

As regards the fragmentation and degradation of plastics accumulated on the ground and on the coasts (which seem less worrying for human health but still to be taken into consideration), suitable collection and recycling policies and procedures would be able to mitigate (and, in some cases, practically eliminate) the related impacts; furthermore, suitable recovery practices and the use of biodegradable plastics may also help in mitigating the impacts related to micro- and nano-plastics.

## Figures and Tables

**Table 1:** Classification based on particle size.

Kind	Size Range	Source
MACROPLASTICS	≥2.5 cm [7,8,9]	“Whole” objects or fragments of packaging and various objects in polymeric material, mainly due to braking processes or direct, intentional release
MESOPLASTICS	5 mm–2.5 cm [7,8,9]	As above
PRIMARY MICROPLASTICS	≤5 mm [8,9], 1–5 mm [7,10]	Scrubbers in cosmetics, liquid soaps, paints, abrasive products; pharmaceuticals; pellets; textile fibers; release of particles and fibers from production and maintenance operations of objects made of polymeric material
SECONDARY MICROPLASTICS	≤5 mm [8,9], 1–5 mm [7,10]	Coming from macroplastics and mesoplastics (but also from primary microplastics) essentially through environmental degradation processes: thermal, thermo-oxidative, photo-oxidative, biological; above all, from UV radiation; also mechanical, like washing and drying of fabrics, or tire abrasion
MINIMICROPLASTICS	1 µm–1 mm [11]	As for microplastics in general (and secondary plastics in particular)
NANOPLASTICS	1 nm–100 nm [7,10,12], <1 µm [11]	According to some: the same of microplastics in general and primary plastics in particular, so much that “paints, adhesives, drugs, electronics, and new 3D printing technologies” are cited [13,14]; according to others: mainly like secondary microplastics, especially washing and drying of fabrics, deterioration of tires and other products such as polystyrene [15,16] or even almost exclusively fabrics and tires [12,17]

**Table 2:** Main sources of Unintentional Microplastics Release (UMR) to the EU environment (data taken from [37] under Creative Commons CC-BY 4.0 License).

Main Sources of UMR to the EU Environment	Lower Estimates (2019—Tonnes/Year)	Higher Estimates (2019—Tonnes/Year)
Paints	231,000	863,000
Tires	360,000	540,000
Pellets	52,140	184,290
Textiles	1649	61,078
Geotextiles	6000	19,750
Detergent capsules	4140	5980

## Data Availability

Not applicable.
